# Stock-outs of essential medicines among community health workers (CHWs) in low- and middle-income countries (LMICs): a systematic literature review of the extent, reasons, and consequences

**DOI:** 10.1186/s12960-022-00755-8

**Published:** 2022-07-15

**Authors:** Abimbola Olaniran, Jane Briggs, Ami Pradhan, Erin Bogue, Benjamin Schreiber, Hannah Sarah Dini, Hitesh Hurkchand, Madeleine Ballard

**Affiliations:** 1 Community Health Impact Coalition, Lagos, Nigeria; 2 Management Sciences for Health, Washington, DC United States of America; 3 New York University, New York, NY United States of America; 4 UNICEF, New York, NY United States of America; 5 Department of Global Health and Health System Design, Arnhold Institute for Global Health, Icahn School of Medicine at Mount Sinai, 1216 5th Ave, New York, NY 10029 United States of America; 6 Community Health Impact Coalition, London, UK

**Keywords:** Community health workers, Frontline health workers, Supply chain, Stock out, Health system, Universal health coverage

## Abstract

**Background:**

This paper explores the extent of community-level stock-out of essential medicines among community health workers (CHWs) in low- and middle-income countries (LMICs) and identifies the reasons for and consequences of essential medicine stock-outs.

**Methods:**

A systematic review was conducted and reported in line with the Preferred Reporting Items for Systematic Reviews and Meta-Analyses (PRISMA) guidelines. Five electronic databases were searched with a prespecified strategy and the grey literature examined, January 2006–March 2021. Papers containing information on (1) the percentage of CHWs stocked out or (2) reasons for stock-outs along the supply chain and consequences of stock-out were included and appraised for risk of bias. Outcomes were quantitative data on the extent of stock-out, summarized using descriptive statistics, and qualitative data regarding reasons for and consequences of stock-outs, analyzed using thematic content analysis and narrative synthesis.

**Results:**

Two reviewers screened 1083 records; 78 evaluations were included. Over the last 15 years, CHWs experienced stock-outs of essential medicines nearly one third of the time and at a significantly (*p* < 0.01) higher rate than the health centers to which they are affiliated (28.93% [CI 95%: 28.79–29.07] vs 9.17% [CI 95%: 8.64–9.70], respectively). A comparison of the period 2006–2015 and 2016–2021 showed a significant (*p* < 0.01) increase in CHW stock-out level from 26.36% [CI 95%: 26.22–26.50] to 48.65% [CI 95%: 48.02–49.28] while that of health centers increased from 7.79% [95% CI 7.16–8.42] to 14.28% [95% CI 11.22–17.34]. Distribution barriers were the most cited reasons for stock-outs. Ultimately, patients were the most affected: stock-outs resulted in out-of-pocket expenses to buy unavailable medicines, poor adherence to medicine regimes, dissatisfaction, and low service utilization.

**Conclusions:**

Community-level stock-out of essential medicines constitutes a serious threat to achieving universal health coverage and equitable improvement of health outcomes. This paper suggests stock-outs are getting worse, and that there are particular barriers at the last mile. There is an urgent need to address the health and non-health system constraints that prevent the essential medicines procured for LMICs by international and national stakeholders from reaching the people who need them the most.

**Supplementary Information:**

The online version contains supplementary material available at 10.1186/s12960-022-00755-8.

## Background

Community health workers (CHWs) are paraprofessionals or lay individuals based in the community who provide health services to urban and rural communities [1]. The role of CHWs is increasingly recognized as key to achieving Universal Health Coverage and pandemic preparedness and response [2, 3]. CHWs continue to play critical roles in reaching communities with essential health services because of their social and geographical proximity to residents and the cost-effectiveness of their services [4–6]. Delivery of Integrated Community Case Management (iCCM), for example, has been shown to reduce child deaths from malaria, pneumonia, and diarrhea by up to 60% [7–11]—but only if CHWs have access to essential supplies. Essential medicines consist of drugs with a potential for safe treatment of priority conditions and these drugs make up the minimum medicine needs for a basic healthcare system [12]. CHWs are typically supplied with these drugs by a primary health center to which they are linked. Systematic and narrative reviews have found that stock-out of such essential medicines is a major hindrance to CHW productivity and motivation [13, 14]. Evidence suggests that stockout of essential medicine may result in poor clinical outcomes, including drug-resistant mutation and increased mortality [15].

Understanding the extent of, reasons for and consequences of stock-outs of essential medicines at the community level is critical for optimizing CHW programs [16]. Some projects, such as Improving Supply Chains for Community Case Management of Pneumonia and Other Common Diseases of Childhood (SC4CCM), implemented by JSI Research & Training Institute, have documented essential medicine stock-outs at the community level in several countries. However, there is no systematic literature review documenting the extent of CHW stock-outs and elucidating reasons for and consequences of community-level stock-out. This paper attempts to address this knowledge gap using a structured approach.

## Methods

We conducted a systematic review of published and grey literature on the extent of, causes of, and consequences of stock-outs at the CHW level and report it here in line with Preferred Reporting Items for Systematic Reviews and Meta-Analyses (PRISMA) guidelines [17]. Where link health center data were available, it was analyzed, as in most settings, these centers are responsible for resupplying products to CHWs.

### Search strategy

The initial search was conducted between August and September 2018 and updated in March 2021. We reviewed peer-reviewed articles and grey literature published in four electronic databases: [1] PubMed, (2) Global Health via Ovid, (3) Web of Science, and (4) Embase via Ovid. In addition, we searched the Google and Google Scholar search engines and the World Health Organization's website for relevant articles during the same period (date last searched and search strategies used for the databases listed above can be found in Additional file 1: Appendix 1).

A team of supply chain (SC) experts was engaged in soliciting evidence-based, unpublished literature. Grey literature was accessed by requesting documents and reports from supply chain organizations (e.g., USAID, WHO, UNICEF VillageReach, Save the Children, JSI, MSH, Federal Ministries of health etc.). Different platforms, such as the Interagency Supply Chain Group (ISG), Child Health Task Force (CHTF), International Association of Public Health Logisticians (IAPHL), the Community Health Community of Practice, etc., were also approached.

### Inclusion and exclusion criteria

In this study, we defined CHWs as paraprofessionals or lay individuals based in the community who provide health services to urban and rural communities [1]. Several definitions of shortage and stock-out have been used in published and grey literature [18]. For this study, we defined stock-out as: *“the complete absence of a required medicine at a storage point or delivery point for at least 1 day*.” A study was included if it contained (a) information on the number or percentage of CHWs stocked out or (b) reasons for CHW stock-outs or (c) consequences of CHW stock-out.

### Selection and data extraction

Relevant data were systematically extracted from selected studies and tabulated in a Microsoft Excel spreadsheet. The following information was extracted from each study: (1) Study characteristics (including author, title, publication date, year of study, method of study, the sample size of CHWs, the sample size of health centers, geographic area, and the list of essential medicines to be stocked by CHWs, (2) Quantification of stock-out (including number and percentage of CHWs or HFs stocked out), (3) Reasons for stock-out and (4) consequences of stock-out.

### Assessment of risk of bias and quality of evidence

The Risk of Bias in Non-Randomized Studies of Interventions (ROBINS-I) assessment tool [19, 20] was applied to quantitative information from the non-randomized studies (observational studies).

This tool comprises seven bias domains: (1) confounding; (2) selection of participants; (3) classification of intervention; (4) deviation from interventions; (5) missing outcome data; (6) measurement of outcomes; and (7) selection of reported result overall. Risk of bias was rated as: ?—no information; 1—low risk; 2—moderate risk; 3—serious risk; 4—critical risk; and not applicable (N/A)—if the study did not consider the criterion. A study was rated low risk of bias if it was ranked low for all domains; at moderate risk if ranked moderate in at least one domain; at serious risk of bias if it was ranked serious in at least one domain; and at critical risk of bias if it was ranked critical in at least one domain. Overall assessment for any single study was not less severe than the most severe assessment allocated for a single domain for that study.

Qualitative studies relevant to the two themes (reason for and consequence of stock-out) were rated using the GRADE Confidence in the Evidence from Reviews of Qualitative research (CERQual) approach to ascertain the confidence level that can be placed in the findings [21]. Rating was based on methodological limitations; relevance (of the findings in the context of the themes), coherence (of the data in explaining the themes) and adequacy of the data (quantity and richness of data that explain the themes). The overall confidence level was rated down by at least one level for each component for which serious concerns were identified. Overall rating was either high, moderate, low, or very low [22].

Two review authors independently rated all the included studies and resolved any disagreement by reciprocal consulting [20].

### Data analysis

We analyzed only articles published since 2006 to ensure that findings are contemporarily relevant. This cutoff date provides 15 years of data to analyze and corresponds to the period in which CHWs gained renewed attention following the WHO declaration of health workforce shortage [23].

We compared the stock-out rate in the period (2006–2015) before the declaration of sustainable development goals (SDG) and after (2016–2021) to assess quantitative changes in the extent of stock-out. Descriptive statistics of stock-out rates among CHWs and health centers were computed based on the numbers reported by various studies. A two-sample test of means was applied to determine whether a statistical link existed between the CHW and health center stock-out rates over the entire period and for each of the two entities (CHWs and health centers) over the pre and post SDG era (see Additional file 1: Appendix 2).

Using thematic content analysis [24], reasons for stock-out were mapped to relevant segments of the supply chain drawn from structural commonalities of supply chains across countries [25, 26]. Consequences of stock-out were inductively organized based on institutions or individuals affected (Additional file 1: Appendix 3). To create a complete record of findings irrespective of publication year, we documented findings from articles published before 2006 (Additional file 1: Appendix 4).

## Results

### Description of included papers

The systematic review builds on an earlier search which included 48 reports, with 34 of these retained after double screening. As shown in the PRISMA diagram (Additional file 1: Appendix 5), our search of both published and unpublished literature resulted in a total of 1896 records. After the deletion of duplicates, 813 records had their titles and abstracts screened, and 71 records from this screening underwent full-text screening. The 44 records found eligible from this screening were added to the 34 records from the previous search to inform this systematic review. Despite not setting a geographical search limit, all 78 articles are from LMICs including 47 from Africa, 10 from Asia and 21 from more than one country. Thirty-eight of these articles were published in the period 2006–2015, 40 published between 2016 and 2021 and six articles (not included in results for lack of contemporary relevance) published before 2006. Articles published between 2006 and 2021 include 28 articles that contain quantified information on CHW stock-out rate and 67 studies with reasons for and consequences of stock-outs at community level with some studies contributing to both quantitative and qualitative findings.

### Quantitative findings: extent of stock-out

Twenty-eight articles involving 62,372 CHWs and 2,383 health centers described community level stock-outs of essential medicines. Overall, 28.93% [CI 95%: 28.79–29.07] of the CHWs and 9.17% [CI 95%: 8.64–9.70] of the health centers experienced stock-outs of essential medicines during the entire review period. A comparison of the period 2006–2015 and 2016–2021 showed that CHW stock-out level increased from 26.36% [CI 95%: 26.22–26.50] to 48.65% [CI 95%: 48.02–49.28] while that of health centers increased from 7.79% [95% CI 7.16–8.42] to 14.28% [95% CI 11.22–17.34].

Hypothesis testing showed a significant difference (*p* < 0.01) in stock-out levels between CHWs and health centers for the entire review period and each of CHWs and health centers between the two comparison periods (Additional file 1: Appendix 2).

### Qualitative findings: reasons for stock-out at different levels of the supply chain

Reasons for stock-out were grouped under four segments of the supply chain drawing from international descriptions of the supply chain [25, 26]. We present these reasons in the order products move from the central level of the supply chain to CHWs: procurement, distribution, storage and community-level stock management. Overall, issues relating to distribution were the most frequently reported.

#### Procurement

Less than half (*n* = 19) of included papers with qualitative findings described procurement challenges as reasons for stock out (Additional file 1: Appendix 3). The challenges include:

- *Financial issues* There were challenges with inadequate funding from limited domestic budgetary allocation, delayed disbursements [27–35] and an overreliance on unpredictable external funding, especially as termination of donor agreement often leads to disruption in supply [33, 35, 36].
  Furthermore, direct financial allocations to health centers often ignore the population size they cater for and the demands of the community health service provision and disbursement are often delayed and lower than the amount allocated [37]*.*
- *Governance and coordination of national procurement* A key reason for stock-out was delays in procurement from lack of or inadequate governance structure [38]. Other governance-related challenges were lengthy and unclear procurement process [27, 33, 34], frequent changes in key leadership positions within the Ministry of Health with resultant delay in obtaining approval to import medicines into the country [39], and delay in receiving international and domestic orders [40, 41].
- *Logistic management* Insufficient medicine procurement at the central store [42–45] was explained by poor forecasting at district and national levels [38] and unanticipated increased demand, especially with an influx of internally displaced persons (IDPs) during crisis situations [29].

#### Distribution

More than half (*n* = 40) of included papers with qualitative findings described distribution challenges as reasons for stock-out (Additional file 1: Appendix 3). The challenges include:

- *Policies* Lack of or delay in implementing formal policies that stipulate the products that CHWs are permitted to manage and dispense, and policies that formally integrate CHWs into the national supply chain, contribute to CHW stock-outs [35, 41, 46–48]. This includes failure to disseminate policies to health centers resulting in refusal or reticence to supply CHWs with stock [27, 49]. Health centers were often considered to be the “last mile”; therefore, CHW stock is, in some cases, compiled with and viewed as health center stock. Furthermore, restrictive guidelines were preventing the distribution of medicines such as zinc supplement to CHWs [50].
- *Logistic management* Logistic-related stock-out was explained by weak supply chain systems [29, 30, 35, 45, 51–53] from complex and multi-level supply chains [35, 37, 54, 55], fragmentation and duplication from stand-alone supply chains for vertical health programs that work independently of the national supply chain [29, 32, 37, 50].
  Consequently, there was mismanagement of supplies [33], including suspected theft of medicines at different levels of the chain [33, 44, 56], insufficient deliveries [44] and delays [57] from central stores.
- *Information management* Poor communication and coordination between different levels of the supply chain made obtaining information to inform supply chain decisions difficult, especially distribution [35, 58]. This included poor visibility of consumption data due to irregular submission of logistics reports of CHW link health center [59] and no system to track that supply reached the last mile [37, 60]. This may explain the frequently used push system of distribution with a fixed supply that is not data-driven, and which ignores increased demand from awareness campaigns and consequently can result in wastage in some centers and shortage in others [35, 44, 61, 62] and expiry of medicine in centers with supply in excess of their demand [49, 50].
- *Transportation *Delays were often reported in transportation from the district to the health center and subsequently the community. The distance and time required to reach the health centers for resupply [27, 32, 38, 42, 53, 60, 63–67] was also an issue, explained in part by difficult road conditions or terrain [29, 32, 33, 35, 54, 55, 57, 60, 66], especially during the rainy season [29, 51, 60] with floods making some places unreachable [29]. Relatedly, insecurity during travel was often a concern, especially in conflict-affected areas [29, 40].
  CHWs had limited motivation to travel to pick up medicines [53] as they often lacked enough time and transport fare for collection [31, 32, 65]. Lack of dedicated funding for collection was limiting product availability at the community level [27, 29, 35, 37, 49, 54, 55, 67–70]. Where third-party logistic companies were considered for distribution to centers including CHW link health centers, engagement was often delayed by bureaucracy [29].
- *Human resource management:*CHW stock-out resulted from lack of responsiveness to stock-out reports by health centers or district stores [69], perhaps due to a lack of technical competence for managing logistics activities [32, 33, 55] and poor supervisory support [59, 68]. While in general CHWs have been found to follow procedures and perform simple tasks correctly given sufficient orientation and supervision, CHWs were not always guaranteed supplies at the link health center, as this was sometimes threatened by “power tussle” and tense relationship between them and health center workers [29]. In addition, link health centers delay in processing CHWs’ refill requests [29], prioritize their own needs over those of CHWs [36] and may use medicines meant for CHWs to top up their supply especially if availability is tracked at health center level but not CHW level [27, 29, 40, 58, 71]. In addition, link health center often experience stock-out too [49, 65, 66], partly explained by over-prescription of free medicines [29] and may be pressured to appropriate CHWs’ stock.

#### Storage

Less than half (*n* = 7) of included papers with qualitative findings describing storage challenges as reasons for stock-out at CHW and health center levels (Additional file 1: Appendix 3). These include limited or inadequate or improper storage space, which led to CHW stock-outs. The issue of inadequate storage space is not unique to CHWs, who typically store supplies in a box in their homes but also relevant for health centers [36, 37, 66, 72]. Inadequate or inefficient use of space at health center often meant they are unable to keep enough stock to resupply CHWs.

The poor storage conditions have tendencies to compromise the stability and potency of medicines [32, 35, 66, 68, 73]. Furthermore, insecure storage space has the potential for theft [35].

#### Community-level stock management

Less than half (n = 29) of included papers with qualitative findings described challenges relating to community-level stock management as reasons for stock-out (Additional file 1: Appendix 3). The challenges include:

- *Human resource management:*Human resource challenges include shortage of trained staff dedicated to stocktaking and forecasting [27, 35, 36, 44, 54], limited training opportunities and supportive supervision on supply chain management at the health center level [30, 32, 42, 43, 50, 54, 60, 74–77]. CHWs with low literacy/numeracy capacity experienced challenges in reporting and submitting data [58]. This challenge was aggravated when they managed many products, leading to inadequacy in stock management [54]. Hence, data from CHWs may be sparse and of low-quality. In addition, complicated, data-intensive stock-taking was unmanageable for the capacity available at health center and time constraints limit the ability of health center staff to adequately manage logistics [37]. Overall, inadequate training, supervision and poor numeracy skills constrain proper data collection, and utilization, which often leads to poor demand-forecasting, and poor data visibility of community-level consumption data for consolidation into district-level quantification and decision making [35, 37, 53, 58, 60, 65, 70, 74, 78]. Data were often not accurate or, where accurate, did not support decision making [29, 70]. At health center and CHW levels, poor data collection and use make it difficult to accurately estimate needs, contributing to expiry of medicines at the community level [33].
- *Logistic management There were often no* standard procedures or formulas for calculating resupply quantities for CHWs and who should be notified if centers are understocked [32, 58, 68, 70]**.** Insufficient amounts of medicines from poor forecasting [29, 40, 41, 45, 69, 71] do not respond to increased demand for services, including the provision of medicines [28]. CHWs and health center staff report to multiple places using lengthy non-standardized forms that are not user-friendly, thereby creating a slow flow of data necessary to inform supply planning [55, 58, 77]. In addition, poor communication between the health center and central store limits effective planning for stock needs [37].

### Qualitative findings: consequences of stock-outs of essential medicines on stakeholders

#### Program

Five articles described consequences of stock-out on the program, including limited program performance and impact [34, 69, 75, 79] with some programs experiencing stalled implementation [29]. Overall, stock-outs reduce acceptability and confidence of the population in CHW programs [69].

#### Health center

Seven articles described how stock-out affect CHW link health centers. These consequences include increased workload (demand) from case referrals that CHWs couldn’t treat due to stock-out [29, 30, 51]. Health centers with stockout function poorly or are unable to provide services [45, 56, 61]. They may be accused of theft by end-users [56], forced to improvise with other (non-ideal) materials when a medicine is unavailable [46].

#### CHWs

Fifteen articles considered the consequences of stock-out on CHWs to include demotivation from community members’ complaints about lack of medicines [47, 57, 69, 80, 81] and consequent loss of reputation and recognition [82], with some CHWs incurring out-of-pocket expenses to preserve reputation [32, 62]. Ultimately, this may result in job attrition [29, 47, 51].

Stock-out at national levels led to CHWs experiencing a long wait for medicine supply after training [27], thereby limiting their service delivery [52, 79, 82–84] and led to depreciation of CHW competency in medicine administration [47].

#### End-users

Twenty-six papers considered the consequences of stock-out on end-users. Community-level stock-outs meant end-users were not offered services and were referred to the health center by CHWs [29, 33, 66, 73, 85]. Some family planning end-users were uncomfortable with these referrals as health center services were not as discreet as those offered by CHWs [66], resulting in change of contraceptive methods, while others stopped usage [62]. End-users self-referred themselves from a health centers without stock to those with stock [86, 87] based on dissatisfaction from consultations without medicines [88], delay in accessing care within the community due to referrals [69], incurring out-of-pocket cost to buy unavailable medicines [29, 46, 57, 66, 89, 90]. End-users of routine medicines had poor compliance to medicine regimens when they lost access to free medicines [30, 90], and others received inappropriate treatment [91], including underdosing with a resultant incomplete recovery and development of medicine resistance [59]. Overall, end-users had a poor perception of and little confidence in CHWs implementing programs with a medicine stock-out. Consequently, this accounted for low utilization of services [29, 30, 40, 47, 51, 55, 62, 75, 79, 88].

Some end-users resorted to alternative vendors (rather than CHWs and health centers) [92] including herbalists, and hawkers [93], which could put them at risk of receiving counterfeit and substandard medicines.

### Risk of bias and quality of evidence

Using ROBINS-I, 12 of the 28 studies with quantitative findings were evaluated to have a serious overall risk of bias, nine moderate, six low and one had no information for assessing risk of bias.

As shown in (Additional file 1: Appendix 6), the “confounding domain” was a significant contributor to the increased risk of bias of the seven domains of ROBINS-I. The major confounding issue being the co-interventions such as community-focused programs that may have improved availability of essential medicines in the intervention communities. Therefore, the extent of stock-out described in this review may be lower than what obtains in many communities in LMICs, and stakeholders may benefit from primary research to assess the level of local stock-out.

Of the 67 studies with qualitative findings, 12 were considered to have high quality of evidence, 48 moderate quality and seven have low quality. The overall CERQual assessment, explained in a Summary of Qualitative Findings table (Additional file 1: Appendix 7), includes a narrative explanation of the CERQual assessment that highlights issues on methodological limitation and adequacy of data. In light of what we can describe as moderate level of evidence, stakeholders may conduct methodologically sound primary research to identify and quantify the context-relevant reasons and consequences of stock-out in their environment which would guide future interventions. Sensitivity analysis was conducted in which studies at high risk of bias were excluded from the analysis. As shown in Table 6b of Additional file 1: Appendix 6, the stockout rate was even higher among CHWs (52.78%) but lower in health facilities (7.14%), suggesting that without co-interventions (the vast majority of studies were downgraded for confounding), CHWs may experience worse stockout rates.

## Discussion

This review explores the extent, reasons, and consequences of community-level stock-out to provide evidence to guide last-mile supply chain strengthening. It shows that most studies focused primarily on sub-Saharan African countries, perhaps reflecting the popularity of community case management approach or uneven burden of stock-out in sub-Saharan Africa. This review indicates that CHWs were out of stock nearly one third of the time and shows a significantly (*p* < 0.01) higher stock-out rate among CHWs (28.93%) in comparison to the health centers to which they are affiliated (9.17%). This may be explained by the fact that CHWs are often not included in health worker registries used for supply forecasting [94] or are last in line to receive essential supplies in the event of unforeseen shortages [95].

Of note, the health center stock-out rate from our review is lower than rates of between 45.60% and 70.60% documented by WHO in their study of public health sector health facilities across 36 LMICs in 2008 [96]. The lower value from our review may be explained (at least in part) by the co-interventions to improve community-level stocks and support from implementing partners.

Worrisome, however, is the increase in stock-out rate during the period 2016–2021 at both CHW and health center levels, despite growing political commitment to CHWs. Increased commitment must translate to tangible interventions and policy reviews to address all the health and non-health system challenges contributing to stock-outs. If left unattended, it could cause severe setbacks to the universal health coverage and equitable achievement of post-2015 agenda.

This review notes problems relating to distribution as a significant cause of stock-out in LMICs and acknowledges that the private sector could play key roles in strengthening this portion of the supply chain [97]. Nonetheless, the reasons for stock-out are multi-dimensional and would require a multi-sectoral and system approach including coordination of government and donor financing, building quantification into the routine health information system and leveraging mobile technology, enacting and governing policies that support last-mile distribution, capacity building, supervision and motivation of the health workforce on supply chain management and design of simplified stock management tools for CHWs [10].

### Study limitations

We contacted several professionals to access unpublished literature to enrich our findings. That said, the included studies have used various reporting metrics to present stock-outs at the CHW and health center levels which limited possibilities for analysis. Standard criteria for reporting stock-out are recommended for future studies.

While we included only articles published from 2006 to 2021, we compensated for this by comparing our findings with the extent of stock-out and common themes in the reasons for and consequences of stock-out in the period pre-dating 2006 and found no remarkable variance (Additional file 1: Appendix 4).

## Conclusions

Community-level stock-out of essential medicines constitute a serious threat to universal health coverage and equitable improvement of health outcomes. This evidence suggests they are getting worse, rather than better and that CHWs and end-users are disproportionately affected. To ensure equitable and sustainable access to essential medicines, there is a need for intensive remediation of the barriers outlined.

## Supplementary Information

**Additional file 1.** Appendices 1–7.

## Data Availability

All data generated or analyzed during this study are included in its additional information file.

## Abbreviations

CHW: Community Health Worker
GRADE: Grading of Recommendations Assessment, Development and Evaluation
IAPHL: International Association of Public Health Logisticians
iCCM: Integrated Community Case Management
ISG: Interagency Supply Chain Group
JSI: John Snow, Inc.
LMICs: Low- and middle-income countries
LMIS: Logistics Management Information System
MSH: Management Sciences for Health
SC4CCM: Improving Supply Chains for Community Case Management of Pneumonia and Other Common Diseases of Childhood
SHWG: Systems for Health Working Group
USAID: United States Agency for International Development
WHO: World Health Organization
