# Supplementary material for: Stock-outs of essential medicines among community health workers (CHWs) in low- and middle-income countries (LMICs): a systematic literature review of the extent, reasons, and consequences
Source: Hum Resour Health. 2022 Jul 15;20:58. doi: 10.1186/s12960-022-00755-8 (PMC9287964; doi:10.1186/s12960-022-00755-8)
Supplement: Supplementary file 1 — Additional file 1. Appendices 1–7. [file 12960_2022_755_MOESM1_ESM.pdf]

Table of Content

Appendix 1: Database search strategies, including search terms
Appendix 2: Calculating means and comparison of means
Appendix 3: Common themes in the reasons for and consequences of stock-out
Appendix 4: Findings from studies predating 2006
Appendix 5: PRISMA diagram
Appendix 6: Risk of bias of the quantitative findings using ROBINS I and sensitivity analysis
Appendix 7: Quality of evidence of the qualitative findings using GRADE CERTQual
Appendix 8: Completed PRISMA checklist

Appendix 1. Database search strategies, including search terms

Table 1: Study databases and search strategy*

Database(s)	Search strategy
PubMed	("community health workers"[MESH] OR community health distributor[Title/Abstract] OR community health surveyors[Title/Abstract] OR community health assistants[Title/Abstract] OR community health promoters[Title/Abstract] OR female community health volunteers[Title/Abstract] OR health extension workers[Title/Abstract] OR health surveillance assistant[Title/Abstract] OR community based volunteers[Title/Abstract] OR lay health workers[Title/Abstract] OR lady health workers[Title/Abstract] OR volunteer health worker[Title/Abstract] OR voluntary health worker[Title/Abstract])<br>AND<br>(products[Title/Abstract] OR product[title/abstract] OR commodities[Title/Abstract] OR commodity[title/abstract] OR supplies[Title/Abstract] OR supply[title/abstract])<br>Restriction applied
Global Health via Ovid	("community health workers" OR "community health distributor" OR "community health surveyors" OR "community health assistants" OR "community health promoters" OR "female community health volunteers" OR "health extension workers" OR "health surveillance assistant" OR "community based volunteers" OR "lay health workers" OR "lady health workers" OR "volunteer health worker" OR "voluntary health worker")<br>AND<br>(products OR product OR commodities OR commodity OR supplies OR supply)<br>Restriction applied
Web of Science	Please see above (Global Health via Ovid strategy)
Embase via OVID	Please see above (Global Health via Ovid strategy)
Google scholars	(community health worker OR community health distributor OR community health promoter OR OR lady health worker OR volunteer health worker) AND<br>(product OR commodity OR supply)

*Date of last search: 22 March 2021

Appendix 2. Calculating means and comparison of means

Table 2: Summary of findings from studies that assessed stock-out of essential medicine

Author	Study design	Focus	Sample size (f)	% Stock-out (x)	fx	f(x-X¯) ^2 [2006-2021]	f(x-X¯) ^2 [2006-2015]	f(x-X¯) ^2 [2016-2021]
Akulayi 2017	Cross-sectional survey	CHWs	1263	55.50	70096.50	891423.41	Not Applicable	59284.39
		Health center	Missing	Missing	Not Applicable	Not Applicable	Not Applicable	Not Applicable
Anand 2020	Cross-sectional survey	CHWs	26	58.00	1508.00	21966.95	Not Applicable	2273.58
		Health center	35	47.70	1669.50	15937.91	Not Applicable	39094.52
Andersson 2013	Cross-sectional survey	CHWs	2591	77.00	199507.00	5986307.82	6644220.76	Not Applicable
		Health center	Missing	Missing	Not Applicable	Not Applicable	Not Applicable	Not Applicable
Bagonza 2014	Cross-sectional survey	CHWs	336	68.00	22848.00	512809.95	582569.36	Not Applicable
		Health center	Missing	Missing	Not Applicable	Not Applicable	Not Applicable	Not Applicable
Bagonza 2015	Cross-sectional survey	CHWs	300	57.50	17250.00	244819.77	290898.17	Not Applicable
		Health center	Missing	Missing	Not Applicable	Not Applicable	Not Applicable	Not Applicable
Brunie 2018	Cross-sectional survey	CHWs	874	64.00	55936.00	1074744.83	Not Applicable	205967.05
		Health center	Missing	Missing	Not Applicable	Not Applicable	Not Applicable	Not Applicable
Callaghan-Koru 2013	Cross-sectional survey	CHWs	131	31.30	4100.30	733.87	3196.06	Not Applicable
		Health center	Missing	Missing	Not Applicable	Not Applicable	Not Applicable	Not Applicable
Chandani 2012	Cross-sectional survey	CHWs	700	41.35	28945.00	107925.01	157276.91	Not Applicable
		Health center	Missing	Missing	Not Applicable	Not Applicable	Not Applicable	Not Applicable
Chadani 2014	Cross-sectional survey	CHWs	700	63.94	44758.00	857836.52	988546.50	Not Applicable
		Health center	Missing	Missing	Not Applicable	Not Applicable	Not Applicable	Not Applicable

Chipukuma 2020	Cross-sectional survey	CHWs	34	74.00	2516.00	69054.77	Not Applicable	21851.28
		Health center	Missing	Missing	Not Applicable	Not Applicable	Not Applicable	Not Applicable
Colin 2018	Cross-sectional survey	CHWs	60	21.00	1260.00	3776.08	Not Applicable	45867.27
		Health center	Missing	Missing	Not Applicable	Not Applicable	Not Applicable	Not Applicable
Cover 2014	Cross-sectional survey	CHWs	35	54.30	1900.50	22521.73	27321.30	Not Applicable
		Health center	23	60.90	1400.70	61550.64	64872.24	Not Applicable
CTwatch Group 2017	Cross-sectional survey	CHWs	145	40.60	5887.00	19736.79	Not Applicable	9393.49
		Health center	298	5.00	1490.00	5178.96	Not Applicable	567754.12
Doherty 2014	Cross-sectional survey	CHWs	44833	20.27	908764.91	3364710.96	1663113.15	Not Applicable
		Health center	Missing	Missing	Not Applicable	Not Applicable	Not Applicable	Not Applicable
Garg 2020	Cross-sectional survey	CHWs	348	12.50	4350.00	93976.66	Not Applicable	454743.30
		Health center	Missing	Missing	Not Applicable	Not Applicable	Not Applicable	Not Applicable
Gilroy 2012	Cross-sectional survey	CHWs	131	31.00	4061.00	559.62	2819.62	Not Applicable
		Health center	Missing	Missing	Not Applicable	Not Applicable	Not Applicable	Not Applicable
Heidkamp 2015	Cross-sectional survey	CHWs	3717	40.00	148680.00	455241.60	691482.79	Not Applicable
		Health center	Missing	Missing	Not Applicable	Not Applicable	Not Applicable	Not Applicable
IRC-Sierra Leone 2018	Unpublished data	CHWs	3385	46.00	155710.00	985975.86	Not Applicable	3406134.78
		Health center	Missing	Missing	Not Applicable	Not Applicable	Not Applicable	Not Applicable
Jarrah 2014	Costing assessment	CHWs	40	38.00	1520.00	3288.32	5419.00	Not Applicable
		Health center	Missing	Missing	Not Applicable	Not Applicable	Not Applicable	Not Applicable
JSI 2010		CHWs	208	50.00	10400.00	92313.08	520000.00	Not Applicable

	Cross-sectional survey	Health center	85	64.00	5440.00	255548.95	268550.37	Not Applicable
Munos 2016	Cross-sectional survey	CHWs	386	91.00	35126.00	1486986.25	Not Applicable	692339.88
		Health center	Missing	82.00	Not Applicable	Not Applicable	Not Applicable	Not Applicable
Nancy 2012	Cross-sectional survey	CHWs	84	88.00	7392.00	293067.16	319150.63	Not Applicable
		Health center	Missing	Missing	Not Applicable	Not Applicable	Not Applicable	Not Applicable
O'Connell 2011	Cross-sectional survey	CHWs	335	66.70	22344.50	477822.64	545133.78	Not Applicable
		Health center	1769	4.40	7783.60	40229.98	20345.31	Not Applicable
Phok 2017	Cross-sectional survey	CHWs	430	25.90	11137.00	3955.96	Not Applicable	222527.82
		Health center	173	23.50	4065.50	35531.21	Not Applicable	14710.74
SC4CCM 2013*	Controlled before and after assessment	CHWs	248	27.00	6696.00	926.78	Not Applicable	116229.98
		Health center	Missing	Missing	Not Applicable	Not Applicable	Not Applicable	Not Applicable
Shieshia 2014	Cross-sectional survey	CHWs	646	9.21	5949.66	251295.28	190017.03	Not Applicable
		Health center	Missing	Missing	Not Applicable	Not Applicable	Not Applicable	Not Applicable
Smith 2013	Cross-sectional survey	CHWs	249	68.00	16932.00	380028.80	431725.51	Not Applicable
		Health center	Missing	Missing	Not Applicable	Not Applicable	Not Applicable	Not Applicable
Williams 2010	Not documented	CHWs	137	66	9042.00	188231.51	215265.35	Not Applicable
		Health center	Missing	Missing	Not Applicable	Not Applicable	Not Applicable	Not Applicable

* The study was drawn from a systematic review, in which unpublished data were included: Leon N, Balakrishna Y, Hohlfeld A, Odendaal WA, Schmidt BM, Zweigenthal V, et al. Routine Health Information System (RHIS) improvements for strengthened health system management. Cochrane Database Syst Rev. 2020/08/18. 2020;8:Cd012012.

Table 3: Calculation of standard deviation

**Drug stock-out**			
		**CHW**	**Health center**
**2006 - 2021**	Sum fx	1804617.37	21849.30
	Sum f	62372	2383
	X‾ = Sum fx / Sum of f	28.93	9.17
	Variance = Sum (f(x-X‾)^2)/ Sum f-1	286.86	173.79
	Std dev. = √variance	16.94	13.18
	No of studies (count)	28	28
**2006 -2015**	Sum fx	1454394.87	14624.30
	Sum of f	55173	1877.00
	X‾= Sum x / Sum of f	26.36	7.79
	Variance = Sum (f(x-xbar)^2)/ Sum f-1	240.67	188.58
	Std dev. = √variance	15.51	13.73
	No of studies (count)	17	17
**2016-2021**	Sum fx	350222.50	7225.00
	Sum f	7199	506
	P= Sum fx / Sum of f	48.65	14.28
	Variance = Sum (f(x-xbar)^2)/ Sum f-1	727.51	1230.81
	Std dev. = √variance	26.97	35.08
	No of studies (count)	11	11

Tables 2 and 3 illustrate how weighted means (X‾) were calculated by multiplying the mean of stock-out with teach study's sample size, summed each of these results, and divided this by the sum of all the sample sizes using the formula **Σfx/Σf**.

To calculate the standard deviation, we used the formula:

$$s = \sqrt{\frac{\sum f(x-\bar{x})^2}{\sum f}}$$

A. Calculating confidence interval of stock-out mean (95%)

Sample size (number of studies) for CHWs or health center is large enough (greater than 30), and we assume that the data came from a normal distribution. Consequently, we use a *Z**-value from the standard normal (*Z*-) distribution as the critical value.
Confidence interval for a single population:

$\bar{X} \pm Z^* \times s/\sqrt{n}$, where

$\bar{X}$ = mean
n = sample size /number of studies
s = standard deviation

Period: 2006 – 2021

a. CHW medicine stock-out

$\bar{X} = 28.93$, $n = 62372$, $s = 16.94$, $Z^* = 1.96$
Confidence interval = $28.93 \pm 1.96\ (16.94/\sqrt{62372})$
Confidence interval = $28.93 \pm (1.96 \times 0.07)$
Confidence interval = 28.93 ± 0.14

b. Health center medicine stock-out

$\bar{X} = 9.17$, $n = 2383$, $s = 13.18$, $Z^* = 1.96$
Confidence interval = $9.17 \pm 1.96\ (13.18/\sqrt{2383})$
Confidence interval = $9.17 \pm 1.96\ (0.27)$
Confidence interval = 9.17 ± 0.53

Period: 2006 - 2015

a. CHW drug stock-out

$\bar{X}$ = 26.36, n = 55173, s = 15.51, Z* = 1.96
Confidence interval = 26.36 ± 1.96 (15.51/√55173)
Confidence interval = 26.36 ± 1.96 (0.07)
Confidence interval = 26.36 ± 0.14

b. Health center drug stock-out

$\bar{X}$ = 7.79, n = 1877.00, s = 13.73, Z* = 1.96
Confidence interval = 7.79 ± 1.96 (13.73/√1877)
Confidence interval = 7.79 ± 1.96 (0.32)
Confidence interval = 7.79 ± 0.63

Period: 2016 - 2021

a. CHW drug stock-out

$\bar{X}$ = 48.65, n = 7199, s = 26.97, Z* = 1.96
Confidence interval = 48.65 ± 1.96 (26.97/√7199)
Confidence interval = 48.65 ± 1.96 (0.32)
Confidence interval = 48.65 ± 0.63

b. Health center drug stock-out

$\bar{X}$ = 14.28, n = 506, s = 35.08, Z* = 1.96
Confidence interval = 14.28 ± 1.96 (35.08/√506)
Confidence interval = 14.28 ± 1.96 (1.56)
Confidence interval = 14.28 ± 3.06

B. Comparison of means of independent samples (CHW vs health center stock out)

Define Null and Alternative Hypotheses

$H_{O:}$ $X_1 = X_2$
$H_{1:}$ $X_1{}^1X_2$

To compare two means, we will examine if:
i. confidence interval of difference includes zero.
ii. p-value relative to $\alpha = 0.05$
iii. if value of Z-score relative to the rejection region.

Confidence interval = $(\bar{X}_1 – X_2) \pm Z^* \sqrt{}([s_1^2/n_1] + [s_2^2/n_2])$
Z-score = $(\bar{X}_1 – X_2) / \sqrt{}([s_1^2/n_1] + [s_2^2/n_2])$

Period 2006 -2021

a. Comparing the means of CHW stock-out with health center stock-out

$X_1 = 28.93$, $X_2 = 9.17$, $n_1 = 62372$, $n_2 = 2383$, $s_1 = 16.94$, $s_2 = 13.18$, $Z^* = 1.96$
Confidence interval = $(28.93– 9.17) \pm 1.96 \sqrt{}([16.94^2/ 62372] + [13.18^2/2383])$
Confidence interval = $19.76 \pm 1.96 \sqrt{}(0.005 + 0.073)$
Confidence interval = $19.76 \pm 1.96 \sqrt{}0.078$
Confidence interval = $19.76 \pm 1.96\ (0.28)$
Confidence interval = 19.76 ± 0.55
Confidence interval = 19.21, 20.31

Interpretation: The percentage of CHWs experiencing stock-out was greater than the percentage of health facilities experiencing stock-out by about 19.21% and 20.31%.

Calculating the Z-score where $(\bar{X}_1 – X_2)$ = 19.76 and $\sqrt{([s_1^2/n_1] + [s_2^2/n_2])}$ = 0.28, drawn from the solution above
Z-score = 19.76 / 0.28 = 70.57
70.57 is greater than critical value of 1.96. Hence, we reject the null hypothesis.

The number 70.57 is too large to appear in Z-table and the vast majority (close to 1) of the area in the normal curve lies to the left of this Z-score.
Therefore p(Z-score) = 1−1.0000 = 0.0000. The p-value = 0.0000

The data provide sufficient evidence, at the 5% level of significance, to conclude that there is a difference between CHW and center level stock-out.

Period 2006- 2015 vs period 2016-2021

a. Comparing difference in CHW stock-out during the two periods.

X_1 = 26.36, X_2 = 48.65, n_1 = 55173, n_2 = 7199, s_1 = 15.51, s_2 = 26.97, Z* = 1.96
Confidence interval = (26.36– 48.65) ± 1.96$\sqrt{([15.51^2/ 55173] + [26.97^2/7199])}$
Confidence interval = (-22.29) ± 1.96$\sqrt{(0.004+ 0.101)}$
Confidence interval = (-22.29) ± 1.96*0.32
Confidence interval = -22.29 ± 0.64
Confidence interval = -22.93, -21.65
Confidence interval does not include zero.

Z statistic where $(\bar{X}_1 – X_2)$ = -22.29 and $\sqrt{([s_1^2/n_1] + [s_2^2/n_2])}$ = 0.32 drawing from the solution above
Z-statistics = -22.29 / 0.32= -69.66
Hence -69.65 is not within the rejection region of -1.96 and + 1.96. Hence, we reject the null hypothesis.

The number -69.65 is too large to appear in Z-table, and the vast majority (close to 1) of the area in the normal curve lies to the right of this Z-score.
Therefore p(Z-score) = 1−1.0000 = 0.0000. The p-value = 0.0000

P-value of 0.0000 is < 0.05. Hence, there is sufficient evidence to reject the null hypothesis.

b. Comparing difference in health center stock-out during the two periods.

$X_1 = 7.79$, $X_2 = 14.28$, $n_1 = 1877$, $n_2 = 506$, $s_1 = 13.73$, $s_2 = 35.08$, $Z^* = 1.96$
Confidence interval = (7.79– 14.28) ± 1.96√ ([13.73^2/ 1877] + [35.08^2/506])
Confidence interval = -6.49 ± 1.96√ ([0.100] + 2.43)
Confidence interval = -6.49 ± 1.96√ 2.53
Confidence interval = -6.49 ± 1.96*1.59
Confidence interval = -6.49 ± 3.12
Confidence interval = -9.61, -3.37

Confidence interval does not include zero. Hence, there is a significant difference between the means of center level drug stock-outs between the periods 2006-2015 and 2016 - 2021 at a 95% level of confidence.

Z statistic where ($\bar{X}_1 - X_2$) = -6.49 and √ ([s_1^2/n_1] + [s_2^2/n_2]) = 1.59 drawing from the solution above
Z-statistics = -6.49 / 1.59 = -4.08
Hence -4.08 is not within the rejection region of < -1.96 and + 1.96. Hence, we reject the null hypothesis.

The number -4.08 is too large to appear in Z-table, and the vast majority (close to 1) of the area in the normal curve lies to the right of this Z-score. Therefore p(Z-score) = 1−1.0000 = 0.0000. The p-value = 0.0000

The data provide sufficient evidence, at the 5% level of significance, to conclude that there is a difference between health center level stock out in the two periods.
P-value of 0.0000 is < 0.05. Hence, there is sufficient evidence to reject the null hypothesis.

Appendix 3. An inductive approach to identifying the common themes in reasons for and consequences of stock-outs

Table 4: Common themes on the reasons and consequences of stock-out

Theme	Sub-theme (vote count)	Code	Reference
**Reasons for stock-out**	**Procurement (19)**	*Financial Issues*	(27,28,100,29–36)
		Governance and coordination	(27,33,34,38–41)
		Logistic management	(29,38,42–45)
	**Distribution (40)**	*Logistic management*	(29,30,53–57,32,33,35,44,45,50–52)
		Policies	(27,35,41,46–50)
		Information management	(35,44,49,50,58–62)
		Transportation	(27,29,51,53–55,58,60,63–66,31,67–70,32,33,35,38,40,42,49)
		Human resource management	(27,29,66,67,69–71,32,33,36,40,49,55,58,59)
	**Storage (7)**		(32,35,36,67,69,72,73)
	**Stock quantification and requisition (29)**	*Human resource management*	(27,29,50,53,54,58,60,66,68,75–77,30,78,32,33,35,36,42–44)

**Theme**	**Sub-theme (vote count)**	**Code**	**Reference**
		Logistic management	(28,29,69,71,32,40,41,45,55,58,59,68)
**Consequences of stock-out on stakeholders**	**Program (5)**	*Poor performance*	(29,34,70,76,79)
		Reduced acceptability	(70)
	**Health center (7)**	*Increased workload from CHW referrals*	(29,30,51)
		Poor performance	(45,56,61)
		Accusation of theft	(56)
		Improvise with other (non-ideal) materials	(46)
	**CHWs (15)**	*Demotivation*	(47,57,70,80,81)
		Poor performance and depreciation in competency	(27,47,52,79,82–84)
		Loss of reputation	(82)
		Incur out-of-pocket cost for purchase	(32,62)
		Job attrition	(29,47,51)
	**End-users (26)**	*Increased referral and consequent delay in accessing care*	(29,33,67,70,73,85–87,101)
		Change or stoppage of family planning method	(62,67)

Theme	Sub-theme (vote count)	Code	Reference
		Poor compliance to drug regimen	(30,89)
		Incur out-of-pocket cost for purchase	(29,46,57,67,89,90).
		Inappropriate treatment with delay in recovery	(59,91)
		Dissatisfaction and poor perception of CHWs and program, and low utilization of services	(29,30,40,47,51,62,76,79,88)(55)
		Use of alternate vendors including herbalists	(92,93)

Appendix 4: Findings from studies predating 2006

Table 5: Findings from studies predating 2006.

First author (year)	Key findings
Afari 1995	Reason for stock out: CHWs were inadequately supervised on community-level stock quantification and requisition.
Andrianarisoa 1994	Consequence of stock-out: Poor utilization of services by end-users
Brieger 1994:	Extent of stock out among CHWs = 66.17%
Jacobson 1991	A consequence of stock-out in which CHWs returned to using unsafe traditional ways when stock-out disrupt service delivery.

Jacobson 1991(b)	A consequence in which there was impaired service delivery
Stekelenburg 2003	Extent of CHW stock out was 37%. Consequences of stock out on CHWs include loss of reputation and recognition and limited service delivery.

Appendix 5: PRISMA diagram

Figure 1: PRISMA diagram

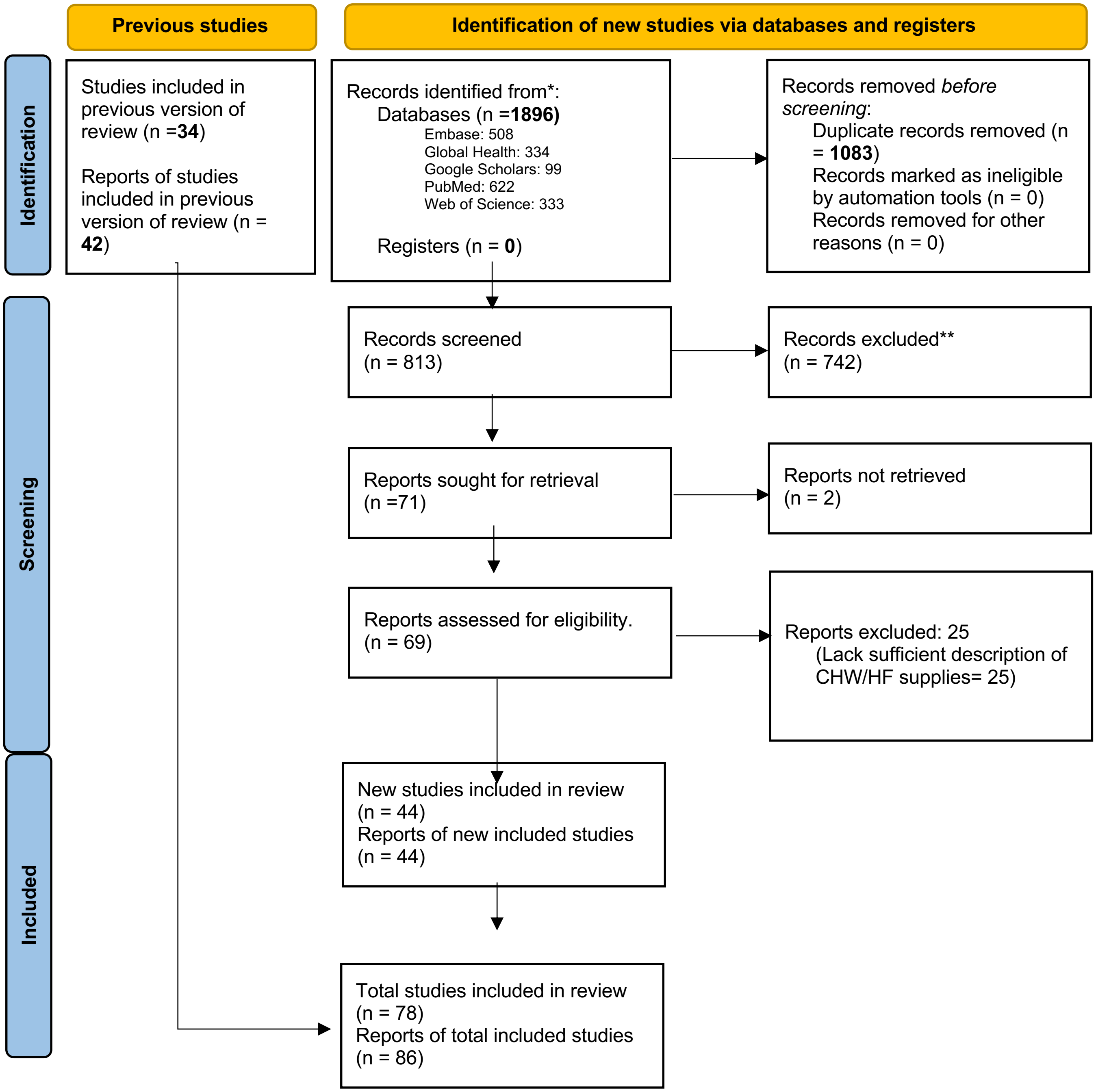

*Consider, if feasible to do so, reporting the number of records identified from each database or register searched (rather than the total number across all databases/registers).

**If automation tools were used, indicate how many records were excluded by a human and how many were excluded by automation tools.

Appendix 6: Risk of bias of quantitative findings

Table 6a: Risk of bias using ROBINS-I domains

First author	Confounding	Selection	Classification	Deviations from intended interventions	Missing data	Measurement of outcomes	Selection of reported result	Overall judgment
Akulayi 2017	1	1	1	N/A	1	1	1	LOW RISK
Anand 2020	1	1	1	N/A	2	1	1	MODERATE
Andersson 2013	3	3	3	3	?	1	1	SERIOUS RISK
Bagonza 2014	1	1	N/A	N/A	1	1	1	LOW RISK
Bagonza 2015	1	2	N/A	N/A	1	1	1	MODERATE RISK
Brunie 2018	1	1	N/A	N/A	2	1	1	MODERATE RISK
Callaghan-Koru 2013	3	1	1	1	1	1	1	SERIOUS RISK
Chandani (Ethiopia) 2012	1	2	1	1	1	1	1	MODERATE RISK
Chandani (Ethiopia) 2014	2	2	1	1	1	1	1	MODERATE RISK
Chipukuma 2020	3	1	1	N/A	1	1	1	SERIOUS RISK
Colin 2018	3	1	1	1	1	1	1	MODERATE RISK
Cover 2014	1	2	N/A	N/A	1	1	1	MODERATE RISK
CTwatch Group, Cyprien Zinsou 2017	1	1	N/A	N/A	1	1	1	LOW RISK
Doherty (Ethiopia) 2014	3	1	1	1	1	1	1	SERIOUS RISK
Garg 2020	3	1	1	1	1	1	1	SERIOUS RISK
Gilroy 2012	1	N/A	N/A	1	1	1	1	LOW RISK
Heidkamp 2015	3	1	1	1	1	1	1	SERIOUS RISK
Jarrah 2014	3	1	1	1	1	1	1	SERIOUS RISK
JSI 2018	3	2	2	1	1	1	1	SERIOUS RISK
Kaludzu 2016	1	2	N/A	N/A	1	1	1	MODERATE RISK
SC4CCM 2013	3	1	1	1	1	1	1	SERIOUS RISK
Munos 2016	2	1	1	1	1	1	1	MODERATE RISK
Nancy 2012	3	2	1	1	1	1	1	SERIOUS RISK
O'Connell 2011	1	1	N/A	N/A	1	1	1	LOW RISK
Phok 2017	1	1	N/A	N/A	1	1	1	LOW RISK
Shieshia 2014	3	1	1	1	1	1	1	SERIOUS RISK
Smith 2013	3	1	1	1	1	1	1	SERIOUS RISK

Williams 2010	?	?	?	?	?	?	?	NO INFORMATION

Figure 2: Overall risk of bias assessment graph

Domain	Assessment	
Confounding		?
Selection bias		?
Classification		?
Deviation from intervention		?
Missing data		?
Measurement of outcomes		?
Selection of reported results		?

Table 6b: Sensitivity analysis excluding studies at high risk of bias

**Drug stock-out**		
	**CHW**	**Facility**
**Sum fx (2006-2021)**	**487809.50**	**16409.3**
**Sum f ((2006-2021)**	**9243**	**2298**
**$\bar{X}$ = Sum fx / Sum of f**	**52.78**	**7.14**
**Variance = Sum (f(x-$\bar{X}$)^2)/ Sum f-1**	**178.16**	**56.66**
**Std dev.**	**13.35**	**7.53**
**No of studies (count)**	**16**	**16**
**Sum fx (2006 -2015)**	**151149**	**9184.30**

**Sum of f (2006 -2015)**	**2674**	**1792.00**
**X̄ = Sum x / Sum of f (2006-2015)**	**56.53**	**5.13**
**Variance = Sum (f(x-xbar)^2)/ Sum f-1**	**140.93**	**40.47**
**Std dev.**	**11.87**	**6.36**
**No of studies (count)**	**8**	**8**
**Sum x (2016 -2021)**	**336660.50**	**7225.00**
**No n (2016 -2021)**	**6569**	**506.00**
**P= Sum fx / Sum of f (2016-2021)**	**51.25**	**14.28**
**Variance = Sum (f(x-xbar)^2)/ Sum f-1**	**689.68**	**1368.80**
**Standard deviation**	**26.26**	**37.00**
**No of studies (count)**	**8**	**8**

Appendix 7: Quality of evidence of qualitative findings

Table 7: Quality of evidence using CERTQual

Author-year	Methodological limitations	Relevance	Coherence	Adequacy of data	Overall judgment (Level of evidence)
Adyya 2018	Insignificant	Moderate	Moderate	Moderate	MODERATE
Afework 2018	Moderate	Moderate	Moderate	Moderate	MODERATE
Aijaz 2014	Moderate	Moderate	Moderate	High	MODERATE
Altaras 2017	Insignificant	High	Moderate	Moderate	MODERATE
Anand 2020	Moderate	High	High	Moderate	MODERATE
Andersson 2010	Significant	High	High	Moderate	LOW
Andersson 2013	Moderate	High	High	Low	LOW
Asweto 2016	Insignificant	High	Moderate	Moderate	MODERATE
Bagonza 2014	Insignificant	Moderate	Moderate	High	MODERATE
Bagonza 2015	Insignificant	High	High	High	HIGH
Blanas 2013	Moderate	Moderate	High	High	MODERATE
Brunie 2018	Moderate	High	Moderate	High	MODERATE
Callaghan-Koru 2013	Insignificant	High	Moderate	High	MODERATE
Chandani 2012	Insignificant	High	High	High	HIGH
Chandani 2013	Significant	High	High	Low	LOW
Chandani (Ethiopia) 2014	Insignificant	High	High	High	HIGH
Chipukuma 2020	Insignificant	High	Moderate	High	MODERATE
Colin 2018	Insignificant	High	Moderate	High	MODERATE
Cover 2014	Insignificant	High	Moderate	High	MODERATE
Doherty (Ethiopia) 2014	Insignificant	High	Moderate	High	MODERATE
Gilroy 2012	Insignificant	Moderate	Moderate	High	MODERATE
HEFDC, Global F-und, UNICEF, WHO(Malawi - Kisunga) 2018	Insignificant	High	Moderate	High	MODERATE
JSI 2018	Significant	High	Moderate	High	LOW
Kavle 2017	Insignificant	High	Moderate	Moderate	MODERATE
Kiwanuka 2017	Insignificant	High	Moderate	High	MODERATE
Kok 2020	Insignificant	High	High	High	HIGH

**Author-year**	**Methodological limitations**	**Relevance**	**Coherence**	**Adequacy of data**	**Overall judgment (Level of evidence)**
Leon 2020	Insignificant	High	Moderate	High	MODERATE
Lufesi 2007	Insignificant	High	High	High	HIGH
Miller 2020	Insignificant	High	Moderate	High	MODERATE
Moti 2019	Insignificant	High	Moderate	High	MODERATE
Mukasa 2017	Insignificant	High	High	High	HIGH
Munos 2016	Insignificant	High	Moderate	High	MODERATE
Naanyu 2020	Insignificant	High	Moderate	High	MODERATE
Nabugoomu 2020	Insignificant	High	Moderate	High	MODERATE
Namazzi 2015	Insignificant	High	Moderate	High	MODERATE
Nancy 2012	Insignificant	High	Moderate	High	MODERATE
Ndou 2013	Insignificant	High	High	High	HIGH
Opwora 2011	Insignificant	Moderate	Moderate	High	MODERATE
Paintain 2014	Insignificant	High	Moderate	Moderate	MODERATE
Panda 2015	Insignificant	High	Moderate	High	MODERATE
Phiri 2017	Insignificant	High	Moderate	High	MODERATE
Printz 2015	Moderate	High	Moderate	High	MODERATE
Pronyk 2016	Moderate	High	Moderate	High	MODERATE
Rao 2013	Significant	High	Moderate	Moderate	LOW
Rawal 2020	Insignificant	High	Moderate	High	MODERATE
Rensburg-Bonthuyzen 2008	Insignificant	High	High	High	HIGH
Ruizendaal 2014	Insignificant	High	Moderate	High	MODERATE
Rutebemberwa 2009	Insignificant	High	Moderate	High	MODERATE
Sakeah 2014	Insignificant	High	Moderate	High	MODERATE
Shaista 2017	Insignificant	High	Moderate	High	MODERATE
Shelley 2016	Insignificant	High	Moderate	High	MODERATE
Shieshia 2014	Insignificant	High	High	High	HIGH

**Author-year**	**Methodological limitations**	**Relevance**	**Coherence**	**Adequacy of data**	**Overall judgment (Level of evidence)**
Smith 2013	Insignificant	High	Moderate	High	MODERATE
Strachan 2014	Insignificant	High	Moderate	High	MODERATE
Sumankuuro 2018	Insignificant	High	Moderate	High	MODERATE
Sunguya 2017	Insignificant	High	Moderate	Moderate	MODERATE
Surakat 2018	Insignificant	High	Moderate	High	MODERATE
Thein 2017	Insignificant	High	Moderate	High	MODERATE
Umulisa 2018	Moderate	Moderate	Moderate	High	MODERATE
UNICEF, WHO, GF. ICCM 2018	Insignificant	High	Moderate	High	MODERATE
USAID Deliver Project. Mozambique 2014	Insignificant	High	High	High	HIGH
Village Reach 2014	Significant	High	High	Moderate	LOW
Village Reach 2015	Insignificant	High	High	High	HIGH
Village Reach 2016	Significant	High	High	Low	LOW
Wagenaar 2014	Insignificant	High	High	High	HIGH
WHO (DRC) 2018	Insignificant	High	Moderate	High	MODERATE
Williams	Moderate	High	Moderate	High	MODERATE

Table 8: Summary of Qualitative Findings table

Summary of review finding	Studies contributing to the review finding	Methodological limitations	Coherence	Adequacy	Relevance	CERQual assessment of confidence in the evidence	Explanation of CERQual assessment
1. Reasons for stock-out relate to procurement, distribution, storage and quantification with distribution being the most cited reason for stock-out. Stakeholders affected were program, health center, CHWs and end-users with the end-users being the most affected.	(27,28,38–47,29,48–57,30,58–67,31,68–73,75–78,32,79–88,33,89,92,93,34–36)	6 articles with significant methodological limitation (unclear description of methodology) 10 articles with moderate methodological limitation. 51 articles with insignificant methodological limitation.	48 articles with moderate coherence. 19 articles with high coherence.	3 articles with low adequacy of data (Poor description of data collection including data saturation). 11 articles with moderate adequacy of data. 53 articles with high adequacy of data.	8 articles with moderate relevance and 59 with high relevance.	Moderate level of confidence	Key concerns relate to poor description of methodology and adequacy of data (data saturation).

Appendix 8: Completed PRISMA checklist

Section and Topic	Item #	Checklist item	Reported on page #
**TITLE**			
Title	1	Identify the report as a systematic review.	1
**ABSTRACT**			
Abstract	2	See the PRISMA 2020 for Abstracts checklist.	2-3
**INTRODUCTION**			
Rationale	3	Describe the rationale for the review in the context of existing knowledge.	4
Objectives	4	Provide an explicit statement of the objective(s) or question(s) the review addresses.	4
**METHODS**			
Eligibility criteria	5	Specify the inclusion and exclusion criteria for the review and how studies were grouped for the syntheses.	5-6
Information sources	6	Specify all databases, registers, websites, organisations, reference lists and other sources searched or consulted to identify studies. Specify the date when each source was last searched or consulted.	5
Search strategy	7	Present the full search strategies for all databases, registers and websites, including any filters and limits used.	Appendix, table 1
Selection process	8	Specify the methods used to decide whether a study met the inclusion criteria of the review, including how many reviewers screened each record and each report retrieved, whether they worked independently, and if applicable, details of automation tools used in the process.	5-6, 7
Data collection process	9	Specify the methods used to collect data from reports, including how many reviewers collected data from each report, whether they worked independently, any processes for obtaining or confirming data from study investigators, and if applicable, details of automation tools used in the process.	6
Data items	10a	List and define all outcomes for which data were sought. Specify whether all results that were compatible with each outcome domain in each study were sought (e.g. for all measures, time points, analyses), and if not, the methods used to decide which results to collect.	4,5
	10b	List and define all other variables for which data were sought (e.g. participant and intervention characteristics, funding sources). Describe any assumptions made about any missing or unclear information.	6
Study risk of bias assessment	11	Specify the methods used to assess risk of bias in the included studies, including details of the tool(s) used, how many reviewers assessed each study and whether they worked independently, and if applicable, details of automation tools used in the process.	6-7
Effect measures	12	Specify for each outcome the effect measure(s) (e.g. risk ratio, mean difference) used in the synthesis or presentation of results.	6
Synthesis methods	13a	Describe the processes used to decide which studies were eligible for each synthesis (e.g. tabulating the study intervention characteristics and comparing against the planned groups for each synthesis (item #5)).	6
	13b	Describe any methods required to prepare the data for presentation or synthesis, such as handling of missing summary statistics, or data conversions.	N/A
	13c	Describe any methods used to tabulate or visually display results of individual studies and syntheses.	6
	13d	Describe any methods used to synthesize results and provide a rationale for the choice(s). If meta-analysis was performed, describe the model(s), method(s) to identify the presence and extent of statistical heterogeneity, and software package(s) used.	7-8
	13e	Describe any methods used to explore possible causes of heterogeneity among study results (e.g. subgroup analysis, meta-regression).	N/A
	13f	Describe any sensitivity analyses conducted to assess robustness of the synthesized results.	N/A

Section and Topic	Item #	Checklist item	Reported on page #
Reporting bias assessment	14	Describe any methods used to assess risk of bias due to missing results in a synthesis (arising from reporting biases).	6-7
Certainty assessment	15	Describe any methods used to assess certainty (or confidence) in the body of evidence for an outcome.	6-7
**RESULTS**			
Study selection	16a	Describe the results of the search and selection process, from the number of records identified in the search to the number of studies included in the review, ideally using a flow diagram.	8-9
	16b	Cite studies that might appear to meet the inclusion criteria, but which were excluded, and explain why they were excluded.	8-9
Study characteristics	17	Cite each included study and present its characteristics.	8-9
Risk of bias in studies	18	Present assessments of risk of bias for each included study.	17
Results of individual studies	19	For all outcomes, present, for each study: (a) summary statistics for each group (where appropriate) and (b) an effect estimate and its precision (e.g. confidence/credible interval), ideally using structured tables or plots.	9-16
Results of syntheses	20a	For each synthesis, briefly summarise the characteristics and risk of bias among contributing studies.	17
	20b	Present results of all statistical syntheses conducted. If meta-analysis was done, present for each the summary estimate and its precision (e.g. confidence/credible interval) and measures of statistical heterogeneity. If comparing groups, describe the direction of the effect.	9
	20c	Present results of all investigations of possible causes of heterogeneity among study results.	N/A
	20d	Present results of all sensitivity analyses conducted to assess the robustness of the synthesized results.	N/A
Reporting biases	21	Present assessments of risk of bias due to missing results (arising from reporting biases) for each synthesis assessed.	17, Appendix 6
Certainty of evidence	22	Present assessments of certainty (or confidence) in the body of evidence for each outcome assessed.	17, Appendixes 6 & 7
**DISCUSSION**			
Discussion	23a	Provide a general interpretation of the results in the context of other evidence.	17-19
	23b	Discuss any limitations of the evidence included in the review.	19
	23c	Discuss any limitations of the review processes used.	19
	23d	Discuss implications of the results for practice, policy, and future research.	17-19
**OTHER INFORMATION**			
Registration and protocol	24a	Provide registration information for the review, including register name and registration number, or state that the review was not registered.	Not registered
	24b	Indicate where the review protocol can be accessed, or state that a protocol was not prepared.	N/A
	24c	Describe and explain any amendments to information provided at registration or in the protocol.	N/A
Support	25	Describe sources of financial or non-financial support for the review, and the role of the funders or sponsors in the review.	20
Competing interests	26	Declare any competing interests of review authors.	20
Availability of data, code and other materials	27	Report which of the following are publicly available and where they can be found: template data collection forms; data extracted from included studies; data used for all analyses; analytic code; any other materials used in the review.	20
